# Amyloid Beta, TNFα and FAIM-L; Approaching New Therapeutic Strategies for AD

**DOI:** 10.3389/fneur.2014.00276

**Published:** 2014-12-18

**Authors:** Paulina Carriba, Joan X. Comella

**Affiliations:** ^1^Institut de Recerca de l’Hospital Universitari de la Vall d’Hebron (VHIR), Barcelona, Spain; ^2^Facultat de Medicina, Departament de Bioquímica i Biologia Molecular, Institut de Neurociències, Universitat Autònoma de Barcelona, Bellaterra, Spain; ^3^Centro de Investigación Biomèdica en Red sobre Enfermedades Neurodegenerativas (CIBERNED), Spain

**Keywords:** soluble amyloid beta, TNFα, neuroinflammation, neurodegeneration, FAIM-L

The aim of this commentary is to complement the review of Franco and Cedazo-Minguez (1).

## Alzheimer’s Disease and Amyloid Beta

Defining characteristics of Alzheimer’s disease (AD) are memory defects, synaptic alterations, presence of neuroinflammatory mediators, and a progressive neurodegeneration. One of the histopathological hallmarks of the disease is the presence of amyloid beta (Aβ) plaques; however, it seems that soluble oligomers, also called Aβ-derive-diffusible-ligands (ADDLs), are the really toxic species involved in the pathogenesis of AD (2). ADDLs are a blend of several sizes of oligomeric Aβ species (3). This suggests that most of the effects on the neurons cannot be attributed to interactions with specific receptors, but rather to interaction and alteration of the proteins and lipids within the cell membranes (4). ADDLs have been detected in AD patients (5), increasing their content with severity (6). Dimers isolated from AD brains impair LTP, enhance LTD, reduce dendritic spines density, and correlate with clinical state (7). Also, they are able to induce hyperphosphorylation of Tau and neuritic dystrophy (8). Soluble oligomers of Aβ are toxic for the neurons (9). They also cause synaptic dysfunction (10) through the activation of caspase-3 (11). Moreover, the inflammatory response characterized by the secretion of various products is initiated by the glial cells when these cells detect Aβ (12). Thus, Aβ appears to be a decisive trigger for the development of this neurodegenerative disorder.

## Neuroinflammation and Neurodegeneration, Two of the Characters in the Progression of the Disease

The neuronal loss observed in the AD brains, as occurs in other neurodegenerative diseases, is produced mainly by apoptosis (13, 14). Sustained neuroinflammatory response contributes to the progression of the disease (15, 16), which ultimately it strengthens the neuronal death (17).

For their physiological importance, both processes are highly regulated; consequently, they can be harmful when deregulated. Apoptosis can be initiated through the mitochondria – intrinsic pathway – or by the stimulation of death receptors (DRs) – extrinsic pathway – [see Ref. (18)]. DRs are cell surface receptors that belong to the TNF super-family. They are able to trigger apoptosis upon ligand binding. DRs and their ligands are expressed physiologically in the brain (19), with important roles in brain development (20, 21) and in cellular homeostasis in adulthood (22). In neurons, in normal conditions, the activation of these receptors does not initiate apoptosis (23, 24). Likewise, inflammation is generally a beneficial physiological response. In fact, it has been described that the initial glial inflammatory response in AD is protective (25, 26).

## TNFα in the Cross-Road between Inflammation and Apoptosis

In brain, TNFα plays a central role in neuroinflammation, apoptosis, and also in the control of the synaptic strength (27, 28). The TNFα gene maps within the class III region of human leukocyte antigen (HLA). Several polymorphisms were detected associated to AD in this region, and systematic meta-analyses concluded that TNFα is a susceptibility gene in the disease (29). High levels of TNFα have been detected in AD patients (30, 31). TNF system has been proposed as a neurotherapeutic target (32), and its role in animal models of AD has been reported (33–35). However, its function in the disease is not clear. It has been described that TNFα is a contributor of the disease (36, 37), although also that it can protect from the Aβ toxicity (38, 39).

TNFα can stimulate two signaling pathways, survival or death (40). The induction of survival pathways depend on NFκB (40) and/or FLIP-L-dependent activation of ERK (41). In normal conditions, TNFα is not toxic for the neurons, indicating that several regulatory proteins prevent the induction of apoptosis at various stages of TNF signaling (42). Expressed exclusively in neurons, the long form of Fas apoptotic inhibitory molecule (FAIM) protein (FAIM-L) is able to regulate the signaling of TNFα. The down-regulation of FAIM-L sensitizes neurons to death induced by TNFα and also by FAS (43). In Parkinson’s disease, it has been proposed that FAIM-L expression could be reduced in dopaminergic neurons, being then this type of neurons more vulnerable to FAS-induced death (44). We have evidences that ADDLs reduce the expression of FAIM-L. The reduction of FAIM-L changes the response mediated by TNFα against the Aβ toxicity, from protection to a contributor in the neuronal death, thus, accelerating the neurodegenerative process (paper under review).

## New Perspectives in Finding Potential Targets

FAIM-L, modulating the function of the TNFα in neurons, would be an example of target molecule able to ameliorate both neurodegeneration and deleterious neuroinflammation. Although speculative, it is possible to hypothesize that the reduction in the neuronal loss would result in an improvement also in the cognition. Aβ is able to cause all the features observed in the disease, thus, targets able to act in more than one of the aspects of the disease would be more useful. However, this type of strategy only would be effective in the prevention of disease progression rather than in the prevention of the disease. Moreover, whereas we do not have good biomarkers for early detection, it seems difficult that potential AD patients (99% of the cases correspond to the non-familiar or sporadic) without any symptom or diagnosis would take drugs to prevent AD in the future, unless these were supplements or healthy habits. Thus, therapies able to prevent the progression of the disease acquire greater relevance.

## Conflict of Interest Statement

The authors declare that the research was conducted in the absence of any commercial or financial relationships that could be construed as a potential conflict of interest.
